# Robotic arm vs. stereotactic frame in deep brain stimulation surgery for movement disorders: a retrospective cohort study

**DOI:** 10.1007/s00701-025-06618-0

**Published:** 2025-08-12

**Authors:** Doriam Perera, Pedro Roldán Ramos, Francesc Valldeoriola, Almudena Sánchez-Gómez, Abel Ferrés, Carlos Pérez-Baldioceda, Gloria Cabrera, Alejandra Mosteiro, Lorena Gómez, Marta Codes, Roberto Manfrellotti, Jordi Rumià

**Affiliations:** 1 Service of Neurosurgery, Hospital Clínic de Barcelona, Barcelona, Catalonia Spain; 2 Service of Neurosurgery, Hospital Militar “Dr Alejandro Dávila Bolaños”, Managua, Nicaragua; 3 Parkinson’s Disease and Movement Disorders Unit, Neurology Service, Institut de Neurociencies, Hospital Clínic of Barcelona, Barcelona, Catalonia Spain; 4 Service of Neurology, Hospital Militar “Dr Alejandro Dávila Bolaños”, Managua, Nicaragua; 5 Departament de Cirurgia i Especialitats Medicoquirúrgiques, Facultat de Medicina i Ciències de la Salut, Universitat de Barcelona (UB), c. Casanova, 143, 08036 Barcelona, Spain; 6 Laboratory of Surgical Neuroanatomy, Department de Anatomía, Facultat de Medicina i Ciències de la Salut, Universitat de Barcelona (UB), c. Casanova, 143, 08036 Barcelona, Spain; 7 Department of Neurosurgery, Hospital Clinic, Departament de Cirurgia i Especialitats Medicoquirúrgiques, Facultat de Medicina i Ciències de la Salut, Universitat de Barcelona (UB), c. Casanova, 143, 08036 Barcelona, Spain

**Keywords:** Deep brain stimulation, Movement disorders, Stereotactic frame, Stereotactic robot

## Abstract

**Background:**

Recently, robotic arms have been incorporated into the implantation of electrodes for deep brain stimulation (DBS).This study aimed to determine the accuracy of brain electrode placement, initial clinical efficacy, and safety profile of the robotic arm Neuromate (Renishaw) compared to a stereotactic frame in movement disorders.

**Methods:**

This study involved two retrospective cohorts: one cohort was operated on using a stereotactic frame and the other with a robotic arm. This study was conducted at Barcelona Hospital Clinic.

**Results:**

Seventy-seven patients were included, of whom 30 underwent surgery using the robot and 47 using a stereotactic frame. The postoperative improvement percentage of the Unified Parkinson’s Disease Rating Scale at 3 months was similar in both groups (robot: 71.4 ± 18 vs. frame: 72.6% ± 17, P = 0.82). There were no significant differences in the perioperative complications (robot: 4% vs. frame: 4.3%, P = 0.93) or in the adverse reactions related to brain stimulation and medical treatment (robot: 18% vs. frame: 25%, P = 0.53). There was a slight improvement in the anatomical-radiological accuracy of brain electrode implantation assisted by the robotic arm, measured using radial error (robot: 1.01 ± 0.5 mm vs. frame: 1.32 ± 0.6 mm, P = 0.03).

**Conclusions:**

Both systems (robotic and stereotactic frame) exhibited similar initial clinical efficacies and safety profiles. The use of the robotic arm Neuromate slightly improved the anatomical-radiological accuracy in the placement of DBS electrodes for movement disorders compared with the stereotactic frame.

**Supplementary information:**

The online version contains supplementary material available at 10.1007/s00701-025-06618-0.

## Introduction

Deep brain stimulation (DBS) is the surgical treatment of choice to significantly enhance the quality of life of patients with certain movement disorders such as Parkinson’s disease, essential tremor, and dystonia [17].

The development and use of the stereotactic frame in functional neurosurgery have allowed the highly precise and safe implantation of DBS electrodes, rendering it as the most appropriate and recommended tool for this purpose [11].

The development of “frameless” implantation systems somewhat revolutionized traditional frame-based systems. However, despite the comparable results, these systems have fallen into disuse [13, 25].

Robotic surgery has gradually been adopted in surgical practice because of its ability to offer enhanced precision and faster recovery of patients, which is supported by ongoing technological advancements. Its integration has enabled increasingly complex procedures to be performed in various medical specialties, with greater safety and efficacy [6, 20, 29].

Recently, international studies have successfully employed different models of stereotactic robots to assist in the placement of DBS electrodes [9, 10, 14, 15, 18, 21, 22, 26, 27, 30, 34, 35].

To the best of our knowledge, prior to this study, no studies have evaluated the effectiveness, safety, and radiological accuracy, these all together, of stereotactic robots compared with stereotactic frames for DBS surgeries.

The objective of the present study was to assess the precision, efficacy, and safety of the Neuromate stereotactic robot (Renishaw) compared with the Leksell G stereotactic frame (Elekta) in a cohort of patients undergoing DBS for Parkinson’s disease.

## Methods and materials

This study has been approved by the Bioethics Committee and is in compliance with international treaties on Bioethics and human rights.

To conduct this study, we followed the recommendations of the Strengthening the Reporting of Observational Studies in Epidemiology (STROBE) methodological guide.[37]

- Description of Groups and Study Variables:

This is a study of two retrospective cohorts: one in which only the Leksell-G stereotactic frame (Elekta®) was used, and the other in which the Neuromate stereotactic robot (Renishaw®) was employed. The study population included patients who underwent surgery for Parkinson’s disease and dystonia at the Neurosurgery Department of the Hospital Clinic of Barcelona between February 1, 2022 and June 1, 2024. The Renishaw® “Neuromate” robotic arm and software began to be used at our center starting in July 2023. Except for the method used to implant the deep brain electrodes (robotic arm vs. stereotactic frame) and the use of two additional intraoperative radiological studies with the O-Arm — performed in nearly 80% of patients in the robotic arm group as a precautionary measure due to the novelty of the technique after cannula placement, which we later found to be unnecessary — all other technical aspects related to diagnosis, preoperative planning, and surgical execution were identical between the two groups, including the neurosurgeons involved. The minimum follow-up period was 3 months to collect information on accuracy, safety, and initial clinical efficacy.

The primary outcome was radiological accuracy measured using radial and vector (Euclidean) errors. Secondary outcomes included improvement in points and percentages on the Unified Parkinson’s Disease Rating Scale (UPDRS-III), postoperative improvement in the total equivalent dose of levodopa, surgical time, presence of significant clinical improvement, surgical complications, radial deviation ≥ 2 mm, and pneumocephalus. Data were retrospectively collected from the electronic medical records of each patient, and pre- and postoperative radiological images (brain computed tomography scans and magnetic resonance imaging) were obtained and analyzed using the Brainlab® automatic segmentation software “Elements” to evaluate radiological accuracy.

b) Anatomical Radiological Accuracy:

The radiological errors measured were radial and vector (or Euclidean) errors. Radial error refers to the distance in millimeters measured from the original DBS electrode placement plan to the actual implanted electrode in the brain, measured at the midpoint of the anatomical target in the axial plane. The radial error considers only the electrode deviation in a single plane (axial). The vector error was measured from the upper edge of the first contact of the planned electrode to the first contact of the implanted electrode. Vector error considers the deviation in three planes (X, Y, and Z) and is a three-dimensional (3D) distance.

- Statistical Analysis:

Statistical analysis began with a comparison of confounding variables between the study groups (Table 1). Statistical significance was defined with a P ≤ 0.05 in any two-tailed statistical test. Clinical significance (effect size) was defined as a percentage difference of greater than or equal to 20% in any analytical comparison. Analysis of the primary outcome was conducted using univariate Student’s t-test. For secondary outcomes, Student’s t-test was used for numerical variables, and the chi-square test was used for categorical variables.

**Table 1:** Characteristics of the study groups; DBS: deep brain stimulation

	Robot DBS surgery		Percentage difference		*P*
	Yes (*n* = 30)	No (*n* = 47) (Frame)			
Mean age (CI 95%)	62 ± 10	59 ± 8	4.8%		0.2
Mean presurgical UPDRS III	34 ± 12	34.9 ± 13	2.5%		0.98
Time on treatment for Parkinson’s disease (years)	9.6 ± 3.6	9.3 ± 3.1	3.1%		0.67
Equivalent dose of preoperative levodopa (mg)	991 ± 376	879 ± 405	11.3%		0.26
Classification by Hoehn and Yahr stages			**Total**	Percentage difference	P
1.0 Unilateral disease	1 (3.3%)	4 (8.5%)	5 (6.5%)	61%	0.36
2–3 Mild to moderate bilateral involvement	20 (66.7%)	38 (81%)	58 (75.3%)	17.6%	0.15
4.0 Severe disability	1 (3.3%)	1 (2.1%)	2 (2.6%)	36%	0.74
Not specified	8 (26.7%)	4 (8.5%)	12 (15.6%)	68%	0.03
Total	30	47	77		
Parkinson’s disease motor phenotype					
Akineto-rigid	12 (40%)	20 (42.5%)	32 (42%)	5.8%	0.82
Dominant tremor	6 (20%)	12 (25.5%)	18 (23%)	21%	0.57
Gait disorder–postural instability	0 (0%)	1 (2.1%)	1 (1.3%)	100%	0.42
Mixed	4 (13.3%)	12 (25.5%)	16 (20.7%)	47.8%	0.19
Not specified	8 (26.6%)	2 (4.2%)	10 (13%)	84.2%	0.004
Total	30	47	77

b) Surgical Technique:

Electrode implantation was performed under general anesthesia without microelectrode recording using direct targeting and intraoperative verification with an O-arm, a technique previously described by our group [31]. For implantation in the study group, a Neuromate robotic arm and software (Renishaw) were used.

As this was a non-experimental, retrospective study that merely reflected routine clinical practice, it was not necessary to obtain informed consent from the patients in the study. All data presented herein, including the supplementary material, were anonymous, and the confidentiality of all patients was maintained.

## Results

We studied 77 patients, of whom 74 underwent surgery for Parkinson’s disease, two for dystonia, and one for essential tremor. The stereotactic frame group included 47 patients and the robotic group comprised 30 patients.

Table 1 presents the main clinical characteristics and confounding variables in both study groups (frame vs. Robot). Overall, no significant differences were found in the primary confounding variables, except that in the group of patients operated on with the robotic arm, there were more cases in which the severity of Parkinson’s disease (Hoehn and Yahr stages) or the motor phenotype of Parkinson’s disease was not described in the clinical records.

The variable that did show a significant difference in favor of the surgeries performed with the stereotactic frame was the surgical time (robot: 3.8 ± 0.9 h vs. frame: 3.2 ± 0.6 h, P = 0.004) (Table 1).

### Anatomical-Radiological Precision in Brain Electrode Placement

Table 2 presents the results of the primary (radial and vector errors) and secondary outcomes between the two study groups. There is a slight improvement in anatomical-radiological precision in electrode placement between the two groups, with the radial error using the robotic arm being 1.01 ± 0.5 mm vs. 1.32 ± 0.6 mm with the stereotactic frame (P = 0.03). Similarly, the vector error using the robot was 1.23 ± 0.4 mm, which was lower compared to the use of the stereotactic frame at 1.56 ± 0.5 mm (P = 0.007) (see Supplementary Figure). Severe deviations of radial error in the placement of brain electrodes (equal to or greater than 2 mm) in the robot group were reduced by 81% compared to frame group (3.3% vs 17.7%, P = 0.05).

**Table 2:** Main results between the study groups. CI: confidence interval; UPDRS: Unified Parkinson’s Disease rating scale; DBS: deep brain stimulation

	Robot DBS surgery		Percentage difference	*P*
	Yes (*n* = 30)	No (*n* = 47)		
Radial error (mean, ± SD)	1.01 ± 0.5	1.32 ± 0.6	23.4%	0.03
Vector error (mean, ± SD)	1.23 ± 0.4	1.56 ± 0.5	21.1%	0.007
Difference in UPDRS III scores postoperatively in ON Stim. and OFF Med. (compared to preoperative UPDRS III) (mean, 95% CI)	−24.5 ± 10	−24.67 ± 12	0.6%	0.97
Percentage improvement in postoperative UPDRS III (mean, 95% CI) (compared to preoperative UPDRS III)	71.4% ± 18	72.6% ± 17	1.6%	0.82
Difference in total daily postoperative dose of levodopa equivalent (mean, 95% CI)	−774 ± 443	−667 ± 459	14%	0.39
Surgical time in hours (mean, 95% CI)	3.8 ± 0.9	3.2 ± 0.6	15.7%	0.004
Surgical time in minutes (mean, 95% CI)	233 ± 58	197 ± 40	15.4%	0.004
Pneumocephalus in cc (mean, 95% CI)	3.7 ± 11	1.8 ± 4	51.3%	0.29

In Supplementary Table 1, we describe the average vector deviations of the electrodes overall and separately for both cohorts. Overall, the study showed a tendency for deviation medially (0.26 mm, 95% CI: $-$ 0.22, 0.7), posteriorly ($-$ 0.4 mm, 95% CI: $-$ 0.04, 0.9), and inferiorly ($-$ 0.24 mm, 95% CI: $-$ 1, 0.5).

**Fig. 1:**
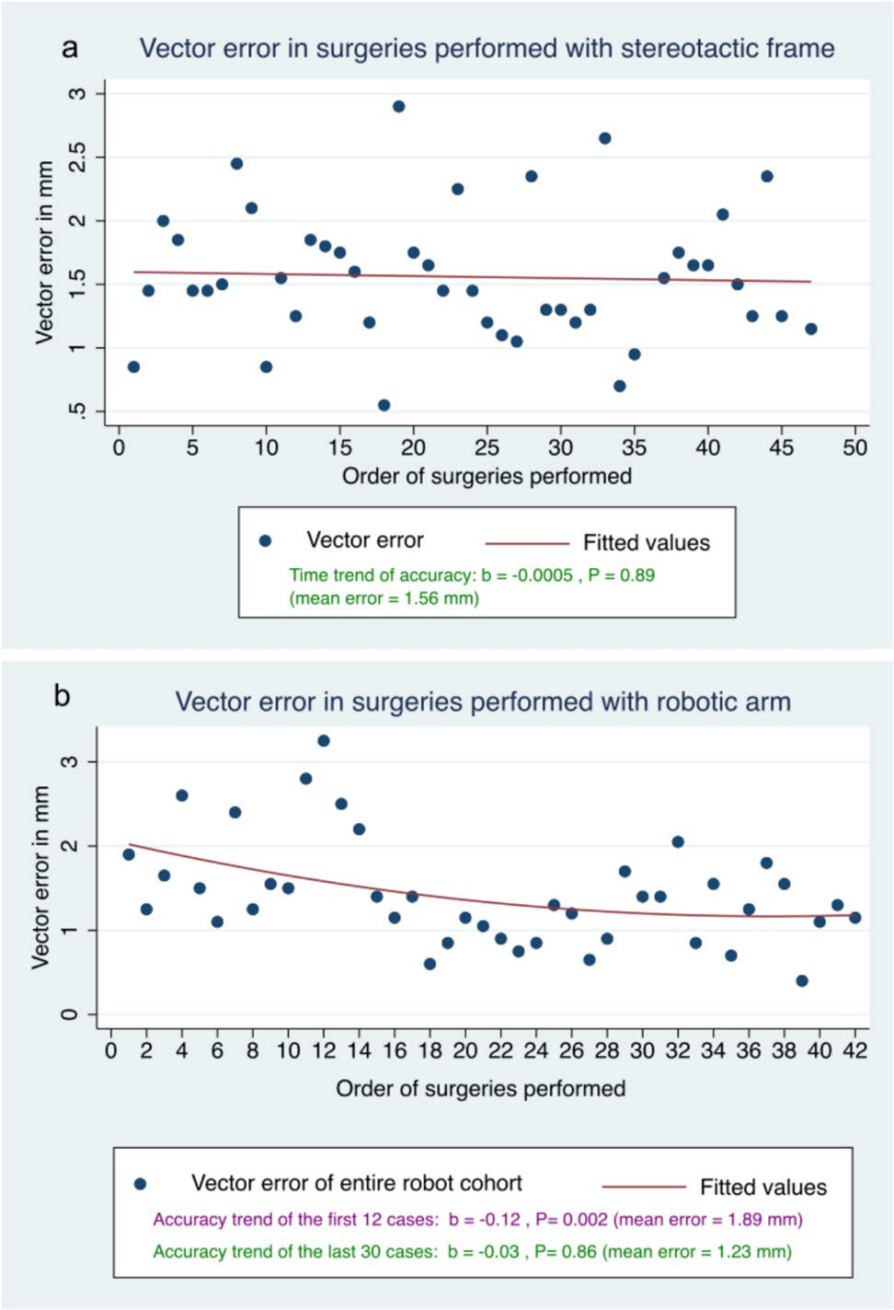
Comparison of vector error in surgeries performed with **A**) a stereotactic frame or **B**) assisted by the robotic arm. In the linear regression model, comparing vectorial error and the order in which each surgery was performed, it can be noted that in patients operated on with the frame, there was no significant change in the precision over time with each surgery (P = 0.89), nor in the last 30 patients operated on with the robot in the cohort included in this study (P = 0.86). The first 12 patients operated on with the robot (not included in the study) showed a significant reduction in error with each new surgery over time (P = 0.002). Accounting for this learning curve effect in the initial robot cases allowed us to create comparable study groups

Initially, 42 patients operated on with the robotic arm were included in the study period, but only the last 30 patients were considered for analysis, as a significant learning curve effect was observed in the first 12 patients, with a tendency toward improved precision that stabilized after the 13th surgery (no further precision changes over time, “plateau state”) (Fig. 1).

In the linear regression model, it can be observed that in the patients operated with the frame, there was no significant change in precision over time with each surgery (P = 0.89), nor in the last 30 patients operated with the robot in the cohort included in this study (P = 0.86). However, the first 12 patients who underwent robotic surgery (not included in the study) showed significant improvement with each new surgery over time (P = 0.002). Accounting for this learning curve effect in the initial robotic cases enabled us to create comparable groups. Therefore, in our study we did not include the first 12 patients we operated on in our service with the robotic arm due to the strong learning curve effect of these first cases.

### Clinical Improvement in Motor Function

Regarding clinical outcomes related to initial clinical effectiveness (at 3 months), no significant differences were found between the two study groups, as clinical improvement in motor function, measured by the UPDRS-III scale, was similar in both groups (percentage of improvement: robot: 71.4% ± 18 vs. frame: 72.6% ± 17, P = 0.82) (Table 2).

The only variable that significantly influenced (reduced) the percentage of improvement in UPDRS-III in both univariate and multivariate analyses was the akinetic-rigid phenotype (P = 0.02) (see Supplementary Table 2).

### Global Impression of Significant Improvement

One of the variables incorporated into the study was the global impression of significant improvement from the patient’s perspective. This significant improvement reflects the symptomatic relief expressed by patients who clearly stated, “that they improved greatly or significantly after surgery and subsequent brain stimulation” or who, in various ways, clearly expressed that the surgery and brain stimulation had significantly improved their quality of life. In cases where patients expressed that they did not experience significant improvement, the improvement was minimal, or where it was unclear or not explicitly mentioned in any clinical note from their electronic medical records whether the patient had experienced significant clinical improvement, these cases were classified as “no significant clinical improvement.”

We decided to include this variable (global impression of clinical improvement) because it could be obtained for 93.5% of patients, complementing the information gathered from the clinical change variable measured using the UPDRS-III scale, as this latter variable could only be measured in 63.6% of patients.

We found that a global impression of significant improvement was achieved in 90% of all patients operated on for DBS, and no significant clinical differences were found between patients operated on with the frame and the robot (frame: 87.2% vs. robot: 96%, RR: 1.1, P = 0.23) (see Supplementary Table 3).

In the univariate and multivariate analysis (see Supplementary Table 4 and 5) of the global impression of significant improvement in both cohorts (frame and robot), only medical treatment equal to or greater than 12 years prior to surgery significantly decreases clinical improvement.

### Complications and Adverse Reactions

No significant differences were observed in the clinical safety variables, as postoperative complications (robot: 4% vs. frame: 4.3%, P = 0.93) and adverse reactions related to stimulation were similar between the study groups (robot: 18% vs. frame: 25%, P = 0.53) (see Supplementary Table 3).

Only one surgical complication occurred in the robotic surgery group: an infection of the implantable pulse generator (neurostimulator). In the frame group, there were two perioperative complications; however, these were medical complications. No deaths occurred during this study. The most frequent adverse reactions related to DBS were dysarthria (9%) and dyskinesia (5%), both of which were transient (see Supplementary Table 6).

### Accuracy According to the Use of Hollow Cannula

**Fig. 2:**
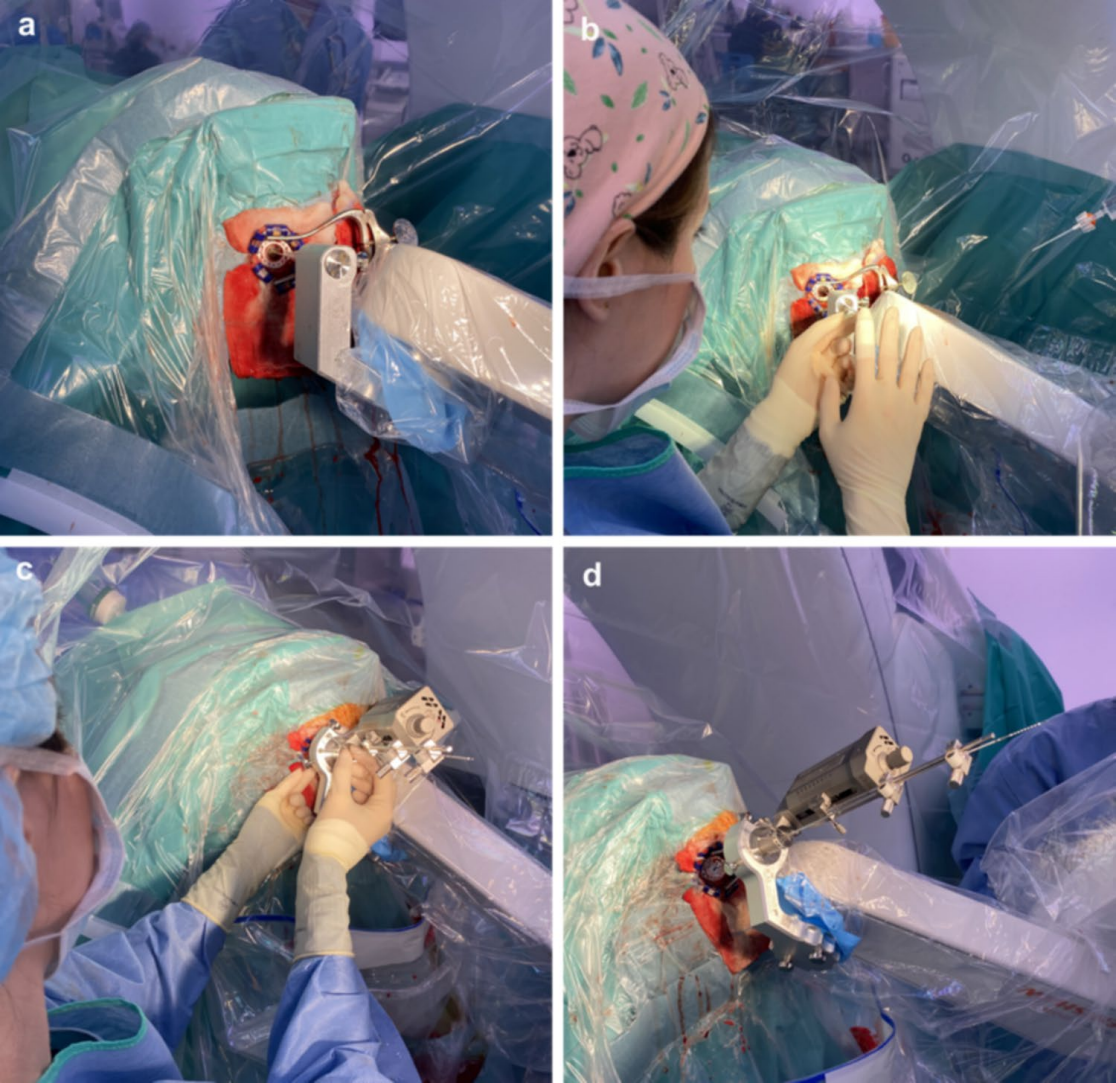
Placement of cannulas and electrodes assisted by robotic arm. **a**) Intracerebral placement of a solid cannula to create a trajectory in the cerebral parenchyma, **b**) placement of a DBS electrode through the trajectory formed by the solid cannula, **c**) and **d**) placement of a hollow cannula and DBS electrode through the hollow cannula. DBS: deep brain stimulation

We conducted a post hoc exploratory analysis of the accuracy of electrode placement according to the use of the cannulas of the robot. This was prompted by the suspicion of one of the two neurosurgeons that the greater deviation of electrodes placed in the early robotic cases was due to the use of a solid cannula with the robotic system instead of a hollow cannula, as is routinely performed with the stereotactic frame for cerebral leads (and electrode) placement. This is because the robotic system lacked a microdrive/electrode holder, which is commonly used with a stereotactic frame, to secure and insert the cannulas. This required the initial introduction of the cannula into the target through the robot’s working channel, followed by removing the cannula and inserting the electrode directly into the target through the channel created by the previously inserted and removed cannula (Fig. 2A, B).

Therefore, in the last 30 robotic cases, one of the two neurosurgeons began placing cerebral leads (and electrodes with a hollow cannula (n = 16), while the other continued using a solid cannula (n = 14). This allowed us to compare the results; however, we found no significant differences in the accuracy of anatomical placement. The vectorial error with the hollow cannula was 1.19 ± 0.40 mm versus 1.28 ± 0.56 mm with the solid cannula (P = 0.6), and the radial error was 1.04 ± 0.52 mm with the hollow cannula versus 0.98 ± 0.49 mm with the solid cannula (P = 0.7) (see Supplementary figure and Fig. 2C and D).

### Sensitivity Analysis Including Patients Excluded in the Analysis

One concern that might remain is what would happen if we included the first 12 patients of the robot cohort in the analysis. We decided to exclude these patients because we observed a significant learning curve effect. In general, when including these patients, the clinical results were similar with the frequency of complications (robot: 7.8% vs. Frame: 4.2%, P = 0.47), adverse reactions (robot: 12.5% ​​vs Frame: 25%), and similar clinical improvement (difference in UPDRS III scores postoperatively: robot: $-$ 21.8 points vs frame: $-$ 24.6 points, P = 0.41). What is very different from the current results is the anatomical-radiological precision, with no significant differences between both study groups, being found with the radial error (robot: 1.18 mm vs Frame: 1.32 mm, P = 0.31) or with the vectorial error (robot: 1.42 mm vs Frame: 1.56 mm, P = 0.26). Regardless, we are willing to share our database (anonymized) with anyone interested in this information.

## Discussion

From our initial experience using a robotic arm for the stereotactic placement of deep brain electrodes for DBS in movement disorders, we found that using the robot offers slight improvement in precision than the stereotactic frame. However, the clinical effectiveness and safety profiles of both technologies were similar.

There are four previous comparative studies that examined the accuracy of the robotic arm versus the stereotactic frame for deep brain electrode placement in DBS for movement disorders [10, 22, 26, 27]. All of these studies showed improved accuracy with the use of the robot compared to the stereotactic frame, with a pooled mean radial error using the robot of 1.13 ± 0.2 mm, which is similar to our study’s radial error of 1.01 ± 0.5 mm. Similarly, in previous studies, the pooled vectorial error was 1.14 ± 0.5 mm (see Supplementary Table 7), comparable to the vectorial error of 1.23 ± 0.4 mm found in our study (Table 2).

The radiological error that showed the greatest improvement in previous research when using the robot compared to the stereotactic frame was the vectorial error, which had a pooled improvement of 20.8%, similar to the 21.1% improvement in vectorial error observed in our study (Table 2). Although previous research also shown an improvement in radial error (by 7%) with the robot, this improvement was greater in our study (23.4%) when the robot was used compared to the stereotactic frame.

The slight improvement in electrode implantation accuracy observed in our study — specifically, a reduction of 0.31 mm in mean radial error and 0.33 mm in mean vector error (Table 3) when using a robotic arm — led a reduction in severe deviations (defined as ≥ 2 mm) in the robotic group, with 81% fewer severe radial deviations compared to the frame-based group (3.3% vs. 17.7%, P = 0.05). This improvement could explain the lower incidence of adverse events related to DBS observed in the robotic group — a 28% reduction (P = 0.53) — likely due to more accurately placed electrodes. However, the lower adverse reactions to stimulation could also be explained by improvements in technology in recent years for programming image-guided deep brain stimulation, which has been implemented at our center by the neurology team of the movement disorders unit [33]. Whether these technical advantages will translate into significant clinical benefits remains to be determined in future studies with larger patient samples [4, 12, 19].

When we began performing the robot-assisted deep brain electrode placement, we observed that the precision was slightly lower than that of the stereotactic frame. One theory we considered was that the type of cannula used with the robot is solid, requiring two brain punctures, which might negatively impact accuracy, as previously reported [3, 4, 8]. However, as previously explained, to resolve this question, in the last 30 robot-assisted surgeries (the group of patients included in our study), half were performed with a hollow cannula and the other half with a solid cannula. We did not find significant differences in the accuracy between the two devices (see the Results section).

The initially lower accuracy observed with the robot proved to be part of the learning curve associated with this new technology, particularly in the first 12 patients who were excluded from our study to make both groups as comparable as possible. At Hospital Clínic de Barcelona, the stereotactic frame has been used for over 20 years for deep brain electrode placement, and no significant reductions in radiological errors have been observed with the current use of the stereotactic frame (Fig. 2).

Regarding motor function improvement, we did not find significant differences between the robot cohort and the stereotactic frame cohorts (percentage improvement in postoperative UPDRS III = robot: 71.4% vs. frame: 72.6%, P = 0.82). This is consistent with two previous comparative studies of robotic arm use, where no significant differences were found in postsurgical percentage improvement in UPDRS III scores [28, 30]. In the percentage improvement in UPDRS III in our study, 71.4%, was higher than in the other two comparative robotic studies, where improvements ranged from 19% to 49.9% [28, 30].

For the patients’ global impression of significant improvement (also known as patient global impression of change), we found no significant differences between the robot cohort and the frame cohorts (robot: 96% vs. frame: 87.2%, P = 0.23). This variable has not been considered in previous comparative studies on robotic DBS surgeries, although it has been studied in other studies on DBS and Parkinson’s disease research [1, 23, 24].

Our results for global patient improvement of 80% and 90% align with other DBS studies for Parkinson’s disease, where percentages of significant patient improvement ranged between 46.6% and 80.9% [1, 23, 24].

We considered it appropriate to include the global patient improvement variable in our analysis to account for patient satisfaction and to complement the motor function improvement analysis with this patient-centered variable. This compensated for the loss of reliability of the postsurgical UPDRS III improvement variable, as we were unable to obtain 36.4% of the retrospective records for this variable.

In analyzing confounding factors affecting motor function measured with UPDRS III (both univariate and multivariate analyses), we found that only the akinetic-rigid phenotype of Parkinson’s significantly affected motor function improvement (see Supplementary Table 2).

Upon reviewing the prior scientific evidence regarding which Parkinson’s disease motor phenotype benefits more from DBS, the results are mixed, with some studies showing a greater response in the akinetic-rigid phenotype and others in the tremor-dominant phenotype [7, 36, 38]. However, studies agree that regardless of the predominant phenotype, tremor is the symptom that improves the most with DBS.

In our analysis of confounding factors for global patient improvement (univariate, multivariate, and sensitivity analyses), we found that only one variable significantly worsened this outcome: Parkinson’s disease duration of more than 12 years (see Supplementary Table 4 and 5).

Although scientific evidence regarding the optimal timing for DBS in Parkinson's varies, an increasing number of centers, including the Hospital Clínic de Barcelona, are opting to operate on patients at earlier stages. This shift follows the success of several randomized clinical trials and cohort studies that operated on patients in earlier disease stages (3–10 years), which led the FDA to approve the use of DBS for Parkinson’s disease patients with at least 4 years of disease duration and the presence of motor complications in November 2015 [5, 16, 32]. For this reason, the average duration of medical treatment for Parkinson's disease in both cohorts in our study was < 10 years (robot: 9.6 years vs frame: 9.3 years).

The safety profiles of the robot and the frame were similar, as we found no significant differences in perioperative complications or adverse effects related to stimulation and medication. While previous studies also did not find significant differences in complications between robotic and frame-based surgeries, our complication rates were slightly lower than in previous comparative studies, where perioperative complications using the robots ranged from 12.9% to 20%. In our study, the surgical complications with the robot were 4% [28, 30].

Although the average surgical time was significantly longer in the robotic group (by approximately 36 min), it is important to note that this increase was primarily due to additional intraoperative imaging. Specifically, 80% of patients in the robotic group underwent two additional O-Arm scans as a safety measure during cannula insertion. In contrast, patients in the frame-based group only had imaging performed after final electrode placement. This additional imaging step (which added approximately 30–40 min) was later deemed unnecessary and subsequently omitted in the most recent robotic cases.

Our study has several limitations, mainly due to its retrospective and non-randomized design, which resulted in incomplete follow-up of some of our variables. This led to missing data for one important clinical variable, the percentage improvement in motor function measured by postsurgical UPDRS III, in 36.4% of patients, which could introduce information bias due to missing data. To a lesser extent, other variables had missing data (see Supplementary Table 8).

Another potential bias in our study was a classification bias, particularly for the clinical variable of global patient improvement, which was assessed retrospectively from clinical records.

One strength of our study is that the main variable, the anatomical-radiological accuracy of electrode placement, had a complete follow-up for most patients, with only two exceptions (97.4% follow-up). Furthermore, radiological accuracy was not influenced by other known confounding variables in our study, making it an ideal variable for monitoring the initial effectiveness of this robotic technology. Another strength was that we achieved near-complete follow-up for perioperative complications in most patients (94.8%).

While robotic arm technology is more expensive — costing twice as much or more, depending on the system — its use may be more cost-effective in high-volume centers treating a broader range of neurological conditions beyond movement disorders. For example, in stereoelectroencephalography (SEEG), robotic systems have demonstrated clear advantages in efficiency compared to traditional stereotactic frames [2, 39]. This is reflected in our own experience: over the past two years, we have routinely used the robotic arm not only for DBS in movement disorders and epilepsy, but also for SEEG electrode placement.

## Conclusions

The use of a robotic arm has improved the anatomical-radiological precision in the placement of DBS electrodes for movement disorders compared to the stereotactic frame. Both systems (robot and frame) had similar initial clinical effectiveness and safety profiles.

## Supplementary information

Below is the link to the electronic supplementary material.

ESM 1 (DOCX 178 KB)

ESM 2 (DOCX 38.7 KB)

ESM 3 (XLS 66.5 KB)

## Data Availability

Database is provided within supplementary data.

## Abbreviations

DBS: Deep brain stimulation
FDA: Food and Drug Administration
UPDRS-III: Unified Parkinson’s Disease Rating Scale
