# Supplementary material for: Robotic arm vs. stereotactic frame in deep brain stimulation surgery for movement disorders: a retrospective cohort study
Source: Acta Neurochir (Wien). 2025 Aug 12;167(1):219. doi: 10.1007/s00701-025-06618-0 (PMC12343702; doi:10.1007/s00701-025-06618-0)
Supplement: Supplementary file 1 — (DOCX 178 KB) [file 701_2025_6618_MOESM1_ESM.docx]

Supplementary figure

**Supplementary Figure**. A) and B) The radiological anatomical accuracy in DBS placement comparing the robotic arm vs. the stereotactic frame, while in C) and D), the accuracy of DBS placement using a hollow cannula vs. a solid cannula is shown. DBS: deep brain stimulation.
