# Supplementary material for: Robotic arm vs. stereotactic frame in deep brain stimulation surgery for movement disorders: a retrospective cohort study
Source: Acta Neurochir (Wien). 2025 Aug 12;167(1):219. doi: 10.1007/s00701-025-06618-0 (PMC12343702; doi:10.1007/s00701-025-06618-0)
Supplement: Supplementary file 2 — (DOCX 38.7 KB) [file 701_2025_6618_MOESM2_ESM.docx]

Supplement 1

**Supplementary Table 1.**Vector deviation in the placement of electrodes for deep brain stimulation. Positive values indicate deviation towards medial, anterior, and superior in the X, Y, and Z planes, respectively. Negative values indicate deviation towards lateral, posterior, and inferior in the X, Y, and Z planes, respectively.

|  | **SURGERY USING ROBOT** | | **Total**  **in mm** | **P** |
| --- | --- | --- | --- | --- |
|  | Yes | No  (Frame) |  |  |
| Medio-lateral deviation (X) in mm | 0.49  ( CI 95% : - 1.2 , 0.2) | 0.33  ( CI 95% : - 0.7 , 0.6) | 0.26  ( CI 95% : - 0.22 , 0.7) | 0.33 |
| Antero-posterior deviation (Y) in mm | - 0.92  ( CI 95% : 0.5 , 1.3) | - 0.04  ( CI 95% : - 0.7 , 0.7) | - 0.4  ( CI 95% : - 0.04 , 0.9) | 0.03 |
| Vertical deviation (Z)  in mm | 0.08  ( CI 95% : - 1.03 , 0.9) | - 0.61  ( CI 95% : - 0.8 , 2) | - 0.24  ( CI 95% : - 1 , 0.5) | 0.36 |

**Supplementary Table 2.** Univariable and multivariable analysis of the percentage improvement in motor function of patients measured using UPDRS-III at 3 months postoperatively.

|  |  | | | **P** | **R^2^** | **P** |
| --- | --- | --- | --- | --- | --- | --- |
|  |  |  | |  |  |  |
| 1. **Univariate analysis**   **Percentage of improvement in UPDRS III postoperatively** | | | | | | |
|  |  |  | |  |  |  |
|  | **Yes** | **No** | |  |  |  |
| Use of robot | 71.4 ± 18 | 72.5 ± 17 | | 0.82 |  |  |
| Akineto: rigid phenotype | 67 ± 18 | 78 ± 13 | | 0.02 |  |  |
| Tremor: dominant phenotype | 79.6 ± 12 | 70.6 ± 18 | | 0.11 |  |  |
| Age equal to or greater than 63 years | 73.8 ± 13 | 72.2 ± 19 | | 0.7 |  |  |
| Radial error equal to or greater than 2 mm | 81.3 ± 9.5 | 71.1 ± 18 | | 0.22 |  |  |
| Vector error equal to or greater than 2 mm | 76.1 ± 11.4 | 71.3 ± 19 | | 0.46 |  |  |
| Medical treatment equal to or greater than 12 years | 76.3 ± 12 | 71.7 ± 18 | | 0.42 |  |  |
| **B) Multivariate analysis**  **Percentage of improvement in UPDRS III postoperatively** | | | | | | |
| Akineto: rigid phenotype | |  | 0.01 | | 0.19 | 0.11 |
| Age equal to or greater than 63 years | |  | 0.52 | |  |  |
| Radial error equal to or greater than 2 mm | |  | 0.22 | |  |  |
| Vector error equal to or greater than 2 mm | |  | 0.85 | |  |  |
| Medical treatment equal to or greater than 12 years | |  | 0.16 | |  |  |

P: level of statistical significance. b: unstandardized coefficient of the multivariable linear regression model. R2: coefficient of determination of the linear regression model. Constant of the model: 75.8.

**Supplementary Table 3. Clinical improvement and complications.** CI: confidence interval; UPDRS: Unified Parkinson's Disease rating scale; DBS: deep brain stimulation.

|  |  | Robot DBS surgery | | | | Total | | RR | P |
| --- | --- | --- | --- | --- | --- | --- | --- | --- | --- |
|  |  | Yes | % | No | % |  |  |  |  |
|  |  |  |  |  |  | n | % |  |  |
| Patient’s global impression of significant improvement at 3 months? | **Yes** | 24 | 96 | 41 | 87.2 | 65 | 90 | 1.1  (CI 95%: 0.9 , 1.2) | 0.23 |
|  | No | 1 | 4 | 6 | 12.7 | 7 | 10 |  |  |
| Improvement greater than or equal to 50% on UPDRS III | Yes | 19 | 90.5 | 25 | 89.3 | 44 | 89.8 | 1.01  (CI 95%: 0.8 , 1.2) | 0.89 |
|  | No | 2 | 9.5 | 3 | 10.7 | 5 | 10.2 |  |  |
| General perioperative complications (surgical and medical) | **Yes** | 1 | 4 | 2 | 4.3 | 3 | 4.1 | 0.9  (CI 95 %: 0.08–9.49) | 0.93 |
|  | No | 25 | 96 | 45 | 95.7 | 70 | 95.9 |  |  |
| Radial deviation greater than 2 mm | Yes | 1 | 3.3 | 8 | 17.7 | 9 | 12 | 0.18  (CI 95%: 0.02 , 1.4) | 0.05 |
|  | No | 29 | 96.7 | 37 | 82.2 | 66 | 88 |  |  |
| Adverse reactions related to stimulation | Yes | 4 | 18 | 11 | 25 | 15 | 23 | 0.72  (CI 95%: 0.2 , 2.0 ) | 0.53 |
|  | No | 18 | 82 | 33 | 75 | 51 | 77 |  |  |

**Supplementary Table 4.** Univariate analysis of confounders with significant clinical improvement in both cohorts (frame and robot).

|  | **Patients’ global impression of significant improvement** | | **P** | **RR** |
| --- | --- | --- | --- | --- |
|  | **Yes** | **No** |  |  |
| **Numeric variables** |  |  |  |  |
| Age  (mean, ± SD) | 59.7 ± 9 | 62.5 ± 7.8 | 0.47 |  |
| Time of medical treatment in years  (mean, ± SD) | 10.6 ± 13 | 13.1 ± 2.4 | 0.63 |  |
| Radial error  (mean ± SD) | 1.21 ± 0.6 | 1.23 ± 0.7 | 0.92 |  |
| Vector error  (mean, ± SD) | 1.43 ± 0.5 | 1.76 ± 0.45 | 0.09 |  |
| Preoperative levodopa equivalent dose  (mean, ± SD) | 916 ± 409 | 876 ± 335 | 0.8 |  |
| Percentage of improvement in UPDRS III after surgery (mean, ± SD) | 72.9 ± 17.6 | 64 ± 18 | 0.32 |  |
| Pneumocephalus (mL) | 2.5 ± 8 | 4.4 ± 8.6 | 0.5 |  |
| **Dichotomous variables** |  |  |  |  |
| Robot Usage ( % ) | 96 |  | 0.23 | 1.1 |
| Akineto-rigid phenotype (%) | 84.3 |  | 0.08 | 0.87 |
| Tremor-dominant phenotype ( % ) | 94.1 |  | 0.57 | 1.05 |
| Mixed Phenotype ( % ) | 100 |  | 0.14 | 1.13 |
| Age equal to or greater than 63 years (%) | 86.6 |  | 0.2 | 0.91 |
| Radial error equal to or greater than 2 mm (%) | 87.5 |  | 0.8 | 0.96 |
| Vector error equal to or greater than 2 mm ( % ) | 83.3 |  | 0.37 | 0.91 |
| Medical treatment equal to or greater than 12 years (%) | 77.7 |  | 0.01 | 0.81 |

**Supplementary Table 5.** Multivariable analysis using multiple logistic regression of variables influencing the global impression of significant patient improvement

| **Variable** | **Odds ratio** | **P** |
| --- | --- | --- |
| Age equal to or greater than 63 years | 0.16 | 0.11 |
| Akineto: rigid phenotype | 0.13 | 0.11 |
| Medical treatment equal to or greater than 12 years | 0.14 | 0.05 |
| Radial error equal to or greater than 2 mm | 0.25 | 0.5 |
| Vector error equal to or greater than 2 mm | 0.68 | 0.79 |

Model value: P = 0.05; pseudo R2 = 0.27; constant: 307.

**Supplementary Table 6.** Description of perioperative complications and adverse reactions related to stimulation.

|  | **Perioperative complications** | **Adverse reactions related to stimulation** |
| --- | --- | --- |
| **Robotic surgery** | - Generator infection (1)   Total = 1 | - Dyskinesias (2) - Dysarthria (2)   Total = 4 |
| Surgery with sterotactic frame | - Heart block requiring cardio-interventional procedure (1) - Urinary retention plus post-renal acute renal failure due to prostatic hyperplasia (1)   Total = 2 | - Dysarthrias (5) - Dyskinesias (2) - Hypophonia (1) - Exacerbation of psychiatric symptoms (2) - Dystonia (1)   Total = 11 |

**Supplementary Table 7.** Radiological errors of previous comparative studies of robot vs. stereotactic frame for deep brain electrode implantation.

|  | Robot | | Frame | |
| --- | --- | --- | --- | --- |
|  | **Vector error** | **Radial error** | **Vector error** | **Radial error** |
| Fenoy 2022 |  | 0.98 |  | 0.74 |
| Ma 2022 |  | 1.28 |  | 1.41 |
| Neudorfer 2018 | 0.76 |  | 1.11 |  |
| Mei 2022 | 1.52 |  | 1.77 |  |
| Pooled Mean | 1.14 | 1.13 | 1.44 | 1.075 |
| Standard deviation | 0.53 | 0.21 | 0.46 | 0.47 |

**Supplementary Table 8.** Complete follow-up of the main outcome variables of the study.

| **Outcome Variable** | **Frequency of complete information** | **Percentage of complete information** | **Risk due to missing data** |
| --- | --- | --- | --- |
| Radial error | 75/ 77 | 97.4 % | Low |
| Vector error | 75/ 77 | 97.4 % | Low |
| UPDRS III scale score | 49/77 | 63.6 % | High |
| Operative time in minutes | 64 / 77 | 83.1 % | Moderate |
| Change in total daily equivalent dose of levodopa | 63 / 77 | 81.8 % | Moderate |
| Impression of significant clinical improvement | 72 / 77 | 93.5 % | Low |
| Presence of early surgical complications | 73 / 77 | 94.8 % | Low |
| Adverse reactions related to stimulation | 66/ 77 | 85.7 % | Moderate |
